# Immune checkpoint inhibitors are effective in the treatment of Epstein-Barr virus-associated gastric cancer: A case report

**DOI:** 10.1097/MD.0000000000033377

**Published:** 2023-03-31

**Authors:** Gaili An, Xin Cheng He, Jun Bai, Jianhua Wang

**Affiliations:** a Department of Internal Medicine Oncology, Shaanxi Provincial People’s Hospital, Xi’an, Shaanxi, China; b Department of General Surgery, Shaanxi Provincial People’s Hospital, Xi’an, Shaanxi, China.

**Keywords:** advanced gastric cancer, Epstein-Barr virus, ICI

## Abstract

Gastric cancer (GC) is one of the most common malignancies globally, and its occurrence and development are associated with genetic, dietary, biological, and immune factors. Epstein-Barr virus-associated gastric cancer (EBVaGC), as a special subtype of GC, has become a research hotspot in recent years. In patients with advanced GC, Epstein-Barr virus infection is closely related to lymph node metastasis, depth of tumor invasion, and poor prognosis. There is great clinical need for a new treatment modality for EBVaGC. Advances in molecular biology and cancer genetics have led to the development of immune checkpoint inhibitors (ICIs); patients treated with ICIs experience clinical benefit and few adverse effects.

**Patient concerns and Diagnoses:**

We report a 31-year-old male with advanced EBVaGC and multiple sites of lymph node metastasis who was intolerant to multiple lines of chemotherapy.

**Interventions and outcome:**

After immune checkpoint inhibitor treatment, both primary and metastatic tumors shrank significantly without noticeable adverse reactions. After 21 months of progression-free status, the patient underwent R0 resection.

**Lessons:**

This case report provides evidence for the use of ICIs in treating EBVaGC. It also shows that detection of Epstein-Barr virus-encoded small nuclear RNA may be a prognostic factor in gastric cancer.

## 1. Introduction

Worldwide, gastric cancer (GC) is the fifth most common cancer and the 4th leading cause of cancer-related death each year.^[1]^ The early stages of GC vary widely across patients, making early diagnosis difficult, and the incidence and mortality of advanced GC are high. In recent years, treatment options based on the molecular properties of tumors are being evaluated in pursuit of achieving improved therapeutic outcomes for GC. Epstein-Barr virus (EBV) is a human herpesvirus that primarily infects epithelial cells, B cells, T cells, and natural killer cells. EBV infection has been associated with several neoplastic and nonneoplastic diseases including nasopharyngeal carcinoma, lymphoma, and GC. In 1993, Tokunaga et al^[2]^ found EBV positivity in GC cells by in situ hybridization and defined it as EBV infection-associated GC. The cancer genome profiling research network in 2014 proposed 4 molecular subtypes of GC: EBV-infected Epstein-Barr virus-associated gastric cancer (EBVaGC), microsatellite instability, genomically stable, and chromosomally unstable.^[3]^ Today, EBVaGC accounts for nearly 10% of all GCs.^[4]^ It is mostly seen in young men, and tends to occur at the junction of esophagus and stomach and remnant stomach. It has a lower clinical stage (moderate to low degree of differentiation) and better prognosis than that of patients negative for EBV infection.^[4–6]^ An analysis of 4599 patients worldwide showed that the median survival of EBVaGC patients was longer than that of EBV-negative GC patients (8.5 vs 5.3 years).^[7]^ Immunotherapy may offer some additional advantage to those patients with EBVaGC. Programmed cell death protein 1 (PD-1) bound to programmed death ligand 1 (PD-L1) can inhibit the activation of cytotoxic T cells and promote tumor cell evasion of the immune system; immune checkpoint inhibitors (ICIs) block the binding of PD-1 to PD-L1 and thereby enhance the body’s antitumor activity. The checkmate 649 study^[8]^ showed that the PD-1 inhibitor nivolumab combined with chemotherapy resulted in superior overall survival compared with chemotherapy alone (14.4 vs 11.1 months). Immunotherapy status is gradually emerging as a prognostic factor in the treatment of advanced GC. Derks et al^[9]^ reported that approximately 15% of EBV (+) gastric cancer cells have gene amplification of chromosome segment 9p24.1, which encodes the ligands for PD-1, suggesting that EBVaGC is more likely to exhibit high expression of PD-1. A 2018 Korean study of 61 patients with advanced GC who were treated with pembrolizumab reported that the objective response rate was significantly higher in PD-L1 (+) patients than in PD-L1 (−) patients (50% vs 0%).^[10]^ Notably, 6 patients with EBVaGC achieved an objective response rate of 100% after immunotherapy. This suggests that ICIs have promising efficacy in advanced EBVaGC. Herein, we report a case of a 31-year-old male patient with advanced GC, whose pathology was suggestive of poorly differentiated adenocarcinoma with EBV infection. The patient was refractory and intolerable to multiple lines of treatment, but developed significant tumor regression when given combined therapy of an antiangiogenic agent with ICI. The patient had no significant adverse effects during immunotherapy. The tumor was successfully transformed from advanced and inoperable to surgically resectable, and a radical gastrectomy was performed after 21 months of ICI treatment. This provides evidence for a new diagnostic criterion and treatment for EBVaGC.

## 2. Case presentation

**Figure 1.**
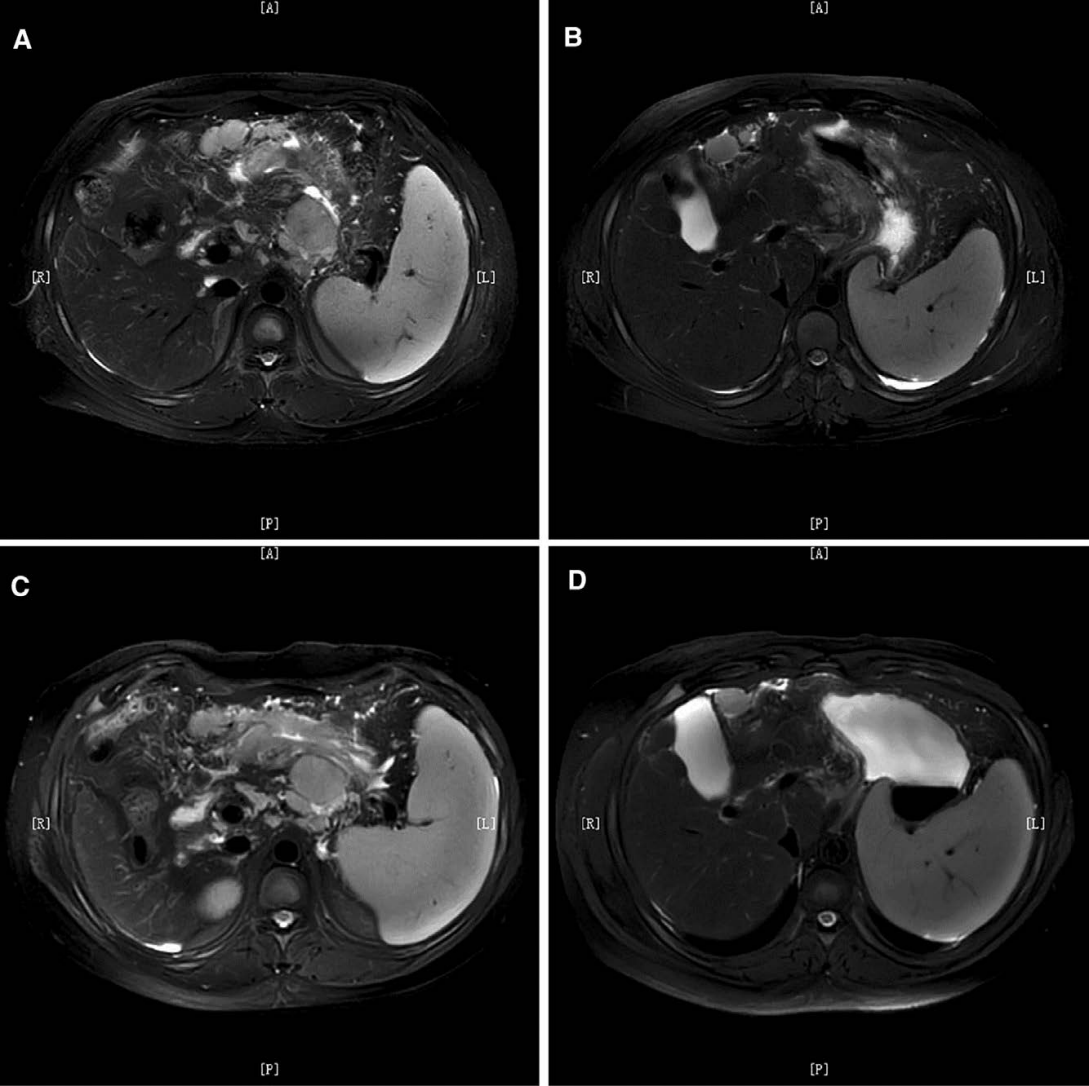
Before treatment, MRI showed uneven thickening of the stomach wall and multiple enlarged lymph nodes in the hepato-gastric space and retroperitoneum, the largest being approximately 4.3 × 4.0 cm (A, B). After 3 cycles of mFOLFOX6 chemotherapy, MRI showed decreased thickness of the gastric wall lesions and some reduction of lymph nodes, the largest being 4.0 × 3.7 cm (C, D).

In June 2020, the patient presented with intermittent mid upper abdominal distension with no previous medical history or relevant family history. Gastroscopy and biopsy revealed poorly differentiated adenocarcinoma of the gastric corpus. Immunohistochemistry showed that the tumor was LCA (−), CD38 (−), CK (+), EMA (+), Ki-67 (50%), c-erb-2 (1 +), Cdx-2 (−), P40 (−), CK8/ 18 (+), p63 (−), and in situ hybridization showed that it was EBER (+). Subsequent abdominal cavity exploration performed in our hospital indicated gastric cancer with multiple lymph node metastases, including retroperitoneal lymph nodes, so radical gastrectomy was not feasible at the time. MRI suggested heterogeneous thickening and abnormal signal shadow in the gastric corpus and wall, enlargement of lymph nodes in the hepato-gastric space and retroperitoneum (maximum size 4.3 × 4.0 cm), enlarged spleen, and a small amount of ascites. Laboratory tests were positive for hepatitis B surface antigen. Three cycles of mFOLFOX6 chemotherapy were given and no significant adverse effects were observed. Post-chemotherapy MRI showed reduced thickness of the gastric wall lesion, some lymph node reduction (maximum size 4.0 × 3.7 cm), and further enlargement of the spleen. The efficacy of first line treatment was evaluated as stable disease (Fig. 1).

As a second line of treatment, cisplatin + capecitabine chemotherapy was combined with concurrent local radiotherapy for the next 2 weeks. Myelosuppression was observed during treatment (platelets at a minimum of 58 * 10^9/L and white blood cells at a minimum of 2.31 * 10^12/L) and was thought to be associated with both radio chemotherapy and hypersplenism. Myelosuppression recovered after treatment with thrombopoietin, avatrombopag, and granulocyte colony-stimulating factor, and splenectomy was performed in October 2020.

**Figure 2.**
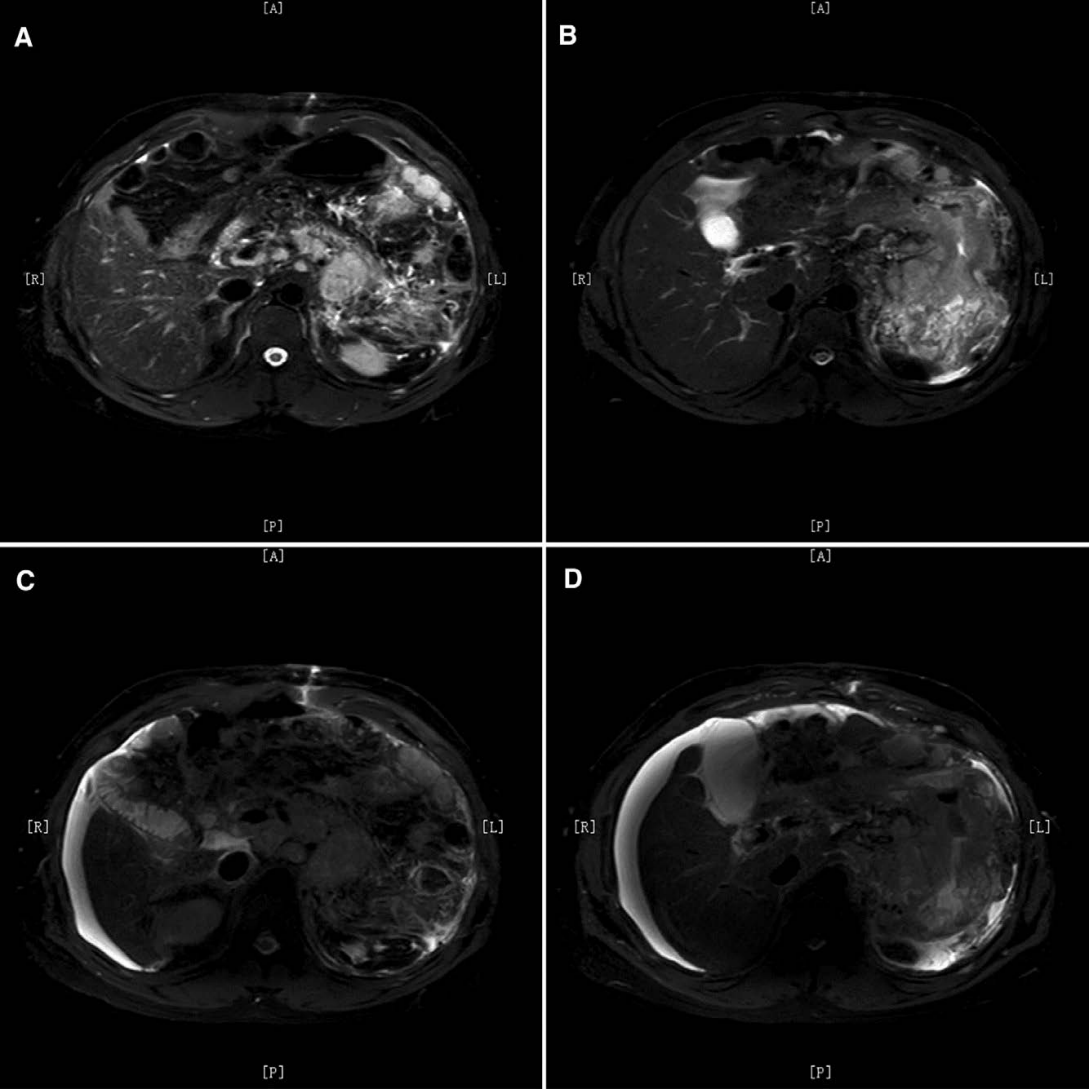
MRI before (A, B) and after (C, D) third line treatment with FLOT chemotherapy showed no change in the stomach wall lesions, significant advancement of multiple lymph node metastases, and unclear boundaries between enlarged lymph nodes and local structures.

**Figure 3.**
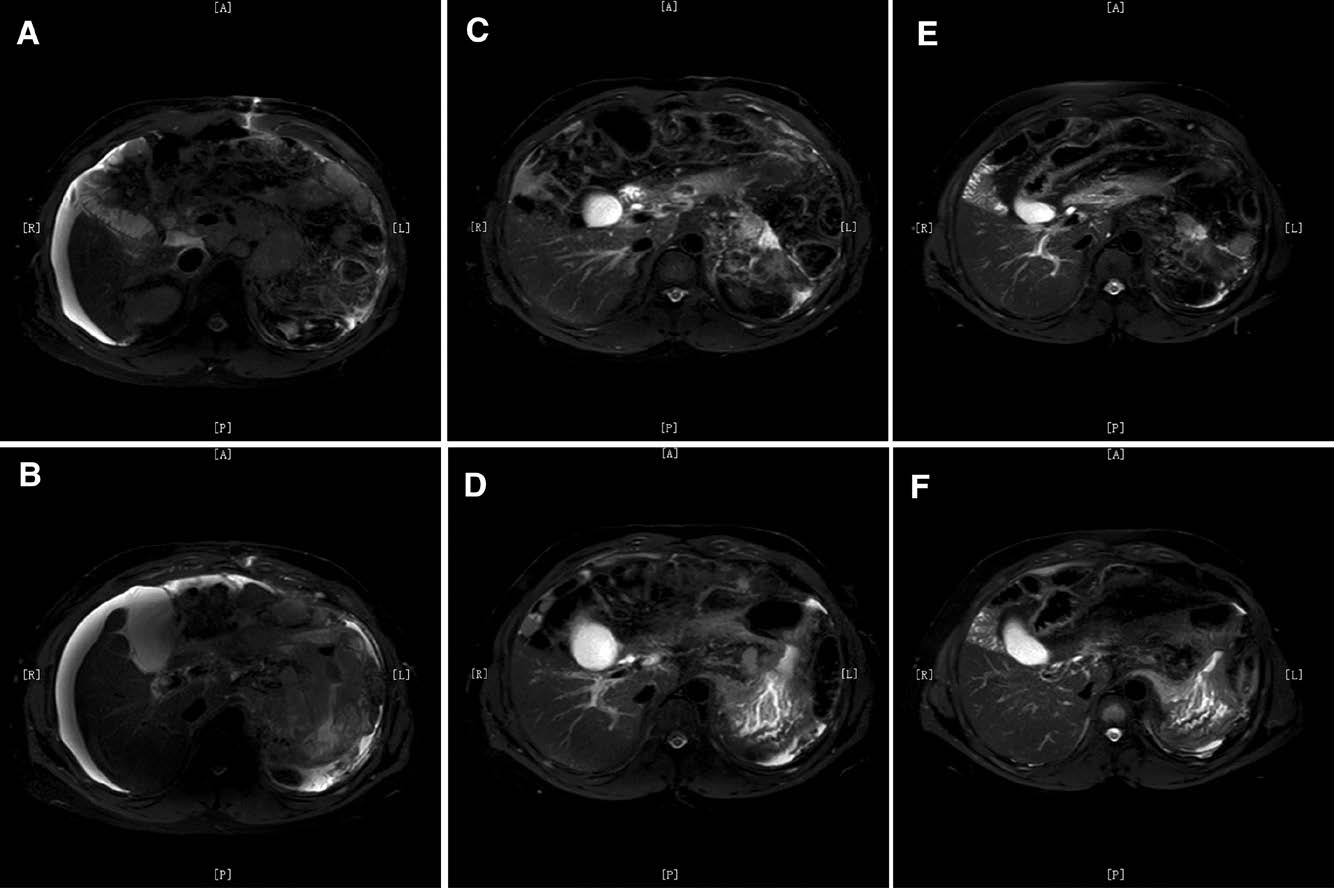
MRI examination before (A, B) and after (C, D) 7 months of ICI treatment showed reduction in the size of stomach wall lesions and lymph nodes, as well as resorption of abdominal fluid. There were no significant additional changes observed after 21 months of treatment (E, F).

**Figure 4.**
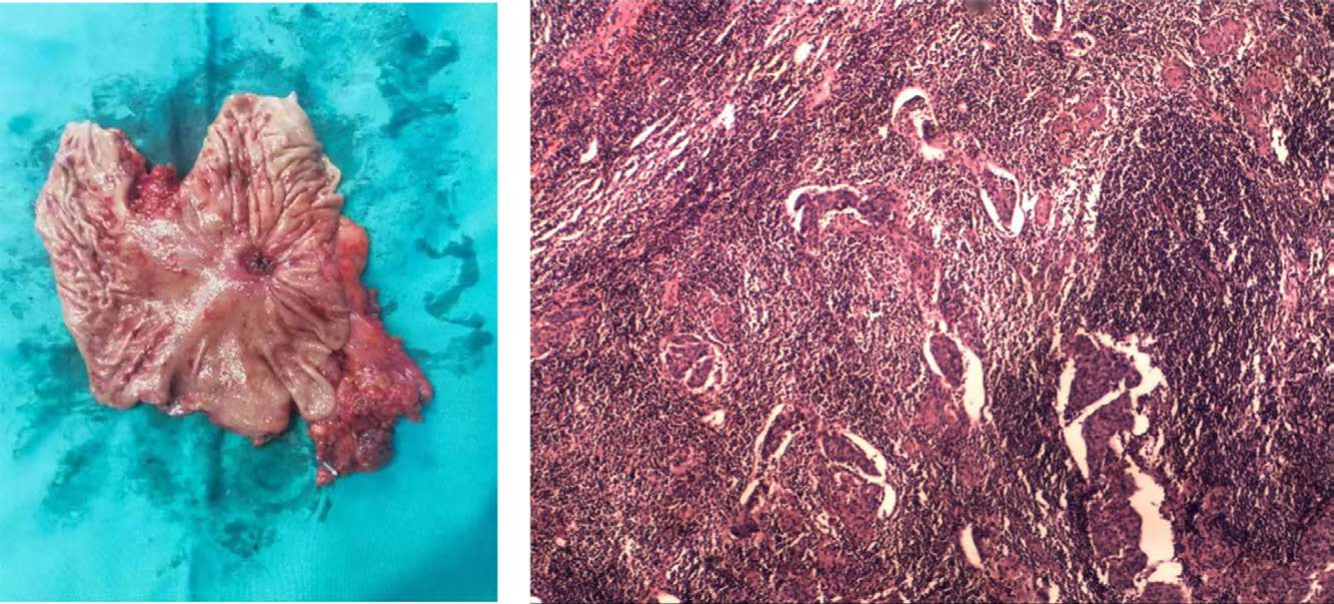
Postoperative pathology showed no residual cancer tissue in the whole stomach, and only 1 of the 21 lymph nodes showed cancer tissue. (A) An open section of the whole stomach shows small ulcerative foci. (B) Hematoxylin-eosin staining of metastatic lymph nodes.

After the myelosuppression had been controlled, 4 cycles of FLOT chemotherapy were administered as a third line of treatment in November 2020. MRI performed in January 2021 showed that lymph nodes were significantly enlarged and indicated progressive disease (Fig. 2). Therefore, the 4th line of treatment was changed to apatinib in combination with the PD-L1 inhibitor sintilimab. Because of intermittent vomiting with blood and significant abdominal pain during treatment, the dosing frequency of apatinib was reduced until symptoms were relieved, and the resulting regimen was used for 26 cycles (21 months). Review of imaging during treatment showed gradual shrinkage of tumor lesions (Fig. 3). PET/CT in September 2022 showed thickening of the gastric wall with hypermetabolism in the antrum of the gastric body, and hypermetabolic enlarged lymph nodes were found in the lesser curvature of the stomach. After comprehensive analysis by a multidisciplinary team, radical gastrectomy was performed in October 2022 to reduce tumor burden. The patient recovered well after surgery, and postoperative pathology showed that there was no residual cancer in the stomach and only 1 of 21 lymph nodes contained cancerous tissue indicative of metastasis (Fig. 4).

## 3. Discussion

Gastroscopy is the primary screening modality for gastric cancer, by which the specific condition of tumors within the stomach can be determined. In situ hybridization is the gold standard for detecting EBV-encoded small RNA to confirm EBVaGC. EBVaGC is histologically similar to lymphoid carcinoma.^[11]^ Its pathological hallmark is a markedly thickened tumor wall, which usually appears ulcerated or sphenoid.^[12]^ It can be classified as lymphoepithelioma-like carcinoma, carcinoma with Crohn disease-like lymphocytic reaction, or conventional adenocarcinoma based on the extent and form of the host immune response, and there is a morphological continuum across these 3 histological subtypes.^[13]^ EBVaGC is predominantly lymphoepithelioma-like and has the best prognosis. The prognosis of patients with EBVaGC is positively correlated with the number and density of lymphocytes infiltrating the tumor tissue, and is also correlated with EBV infection. In the occurrence and development of EBVaGC, immune response and inflammatory reaction play important roles. Recent studies have confirmed that EBV plays an important role in the early stages of gastric carcinogenesis by evading the body’s immune response through the expression of related viral genes and inducing cellular carcinogenesis.^[14]^ Its mechanism of action may be due to the large number of gene promoters that are methylated in EBVaGC. High-throughput sequencing of EBVaGC has shown a high frequency of PIK3C and ARID1A mutations, both of which are associated with tumor development. EBVaGC is also accompanied by high amplification of PD-L1, which can enable tumor cells to undergo immune escape.^[15]^

Immunotherapy has become a common treatment for tumors. PD-1 is a member of the CD28 family that is mainly expressed on the surface of immune cells, thus exerting its immunosuppressive effect. PD-1 has at least 2 ligands (PD-L1 and PD-L2), of which PD-L1 is expressed on a variety of malignant tumor cells.^[16]^ Tumor cells can manipulate their microenvironment to be conducive to tumor growth by activating the PD-1/PD-L1 signaling pathway, which inhibits T-cell activation. Therefore, the immune activity of T cells can be restored by applying PD-1/PD-L1 inhibitors to boost the immune system and thereby kill tumor cells. The PD-1 inhibitor nivolumab showed good efficacy and safety in the Attraction04 study^[17]^: when combined with SOX chemotherapy it showed an overall response rate of 57.1% and progression-free survival (PFS) of 9.7 months, and when combined with CAPOX showed an overall response rate of 76.5% and PFS of 10.6 months. Adverse effects were manageable, suggesting a good safety profile.

PD-L1 expression can be detected in tumor cells of more than half of patients with EBvaGC,^[4]^ and GC cells that are EBV (+) have abundant IFN-γ. IFN-γ released by tumor-infiltrating immune cells can directly induce the expression of PD-L1 by both tumor and immune cells, and EBV also leads to an increase in PD-L1 expression levels via JAK/STAT signaling.^[18]^ It follows that PD-1-driven immune escape may play an important role in EBV, which is also immunosuppressive in the tumor immune microenvironment of EBVaGC. Lv et al^[19]^ reported a patient with EBVaGC (PD-L1 +) who achieved therapeutic effect and a manageable safety profile (PFS was at least 12 months) after treatment with camrelizumab combined with SOX. Pan et al^[20]^ also reported a patient with advanced EBVaGC (high expression of PD-L1 and HER-2) who experienced tumor regression and 28 months of PFS with nivolumab treatment, after not responding to trastuzumab combined with chemotherapy. At present, there are few clinical studies of ICIs for the treatment of EBVaGC, but the existing studies suggest that immunotherapy shows better efficacy and manageable adverse effects in EBVaGC. A retrospective study^[21]^ of 95 EBVaGC patients treated with ICIs showed that the median PFS of EBV + patients were significantly better than those of EBV- patients (8.5 vs 2.0 months; none of the EBV- patients reached 5.0 months). In addition, molecular predictive biomarkers were explored to improve the prediction of ICI efficacy in patients with EBVaGC: density of CTLA-4 and TIM-3 + cells in the tumors of the ICI nonresponder group was significantly higher than that in the ICI responder group. EBVaGC received significant benefit from the suggested ICI combined with CTLA-4 blockade over single immunotherapy, suggesting that tumor mutational burden and SMARCA4 mutations may be useful biomarkers for the prediction of ICI efficacy in patients with EBVaGC.

Combination of ICIs with antiangiogenic therapy is synergistic. The heterogeneous distribution of tumor vessels, tortuosity, and low coverage of endothelial cells make vessels more permeable, the pressure of tissue interstitial fluid rises gradually, and the high pressure further collapses the vessels and reduces perfusion to produce a hypoxic and acidic environment. Together these factors ultimately make the tumor microenvironment less susceptible to immune cell infiltration.^[22]^ antiangiogenic therapy can normalize tumor vessels and convert the immunosuppressive tumor microenvironment into an immune-activated 1 by promoting the accumulation of immune cells, improving the hypoxic environment, and enhancing suppressive cell function. PD-1 promotes the secretion of IFN-γ by cytotoxic T lymphocytes, which then induces tumor cells to secrete VEGF. VEGF in turn promotes PD-1 expression, so it is clear how a combination of antiangiogenic therapy and ICIs can interrupt the cycle, improve the tumor microenvironment, and enhance the activity of T lymphocytes. A phase I study of camrelizumab in combination with apatinib for GC reported promising antitumor effects (objective response rate 17.4%, PFS 2.98 months, and overall survival 11.4 months).^[23]^

## 4. Conclusion

We report a 31-year-old patient with advanced EBVaGC who had poor disease control and experienced severe adverse effects after multiple lines of treatment. ICI treatment combined with apatinib resulted in significant tumor regression and controllable adverse reactions, demonstrating good treatment effect. According to Response Evaluation Criteria in Solid Tumors version 1.1, efficacy was evaluated as partial response. After 21 months of treatment without disease progression, radical gastrectomy was recommended to reduce tumor burden, and was performed successfully. The patient recovered in good condition.

EBV infection rate is high in the population and is closely related to the occurrence and development of some GC. Although infection is latent in the long-term, it remains a potential carcinogenic factor that should not be ignored. This report confirms that ICIs may have significant efficacy in EBVaGC, suggesting them as potential treatments for such patients.

## Author contributions

**Data curation:** Gaili An.

**Formal analysis:** Gaili An.

**Funding acquisition:** Jianhua Wang.

**Investigation:** Gaili An, Jun Bai.

**Validation:** Jun Bai, Jianhua Wang.

**Writing – original draft:** Gaili An, Xin Cheng He.
